# Development and Validation of Unplanned Extubation Prediction Models Using Intensive Care Unit Data: Retrospective, Comparative, Machine Learning Study

**DOI:** 10.2196/23508

**Published:** 2021-08-11

**Authors:** Sujeong Hur, Ji Young Min, Junsang Yoo, Kyunga Kim, Chi Ryang Chung, Patricia C Dykes, Won Chul Cha

**Affiliations:** 1 Department of Digital Health Samsung Advanced Institute for Health Sciences & Technology Sungkyunkwan University Seoul Republic of Korea; 2 Department of Patient Experience Management Samsung Medical Center Seoul Republic of Korea; 3 Department of Nursing College of Nursing Sahmyook University Seoul Republic of Korea; 4 Biomedical Statistics Center Research Institute for Future Medicine Samsung Medical Center Seoul Republic of Korea; 5 Department of Critical Care Medicine Samsung Medical Center Sungkyunkwan University School of Medicine Seoul Republic of Korea; 6 Department of Medicine Samsung Medical Center Sungkyunkwan University School of Medicine Seoul Republic of Korea; 7 Division of General Internal Medicine and Primary Care Brigham and Women’s Hospital Harvard Medical School Boston, MA United States; 8 Department of Emergency Medicine Samsung Medical Center Sungkyunkwan University School of Medicine Seoul Republic of Korea; 9 Digital Innovation Center Samsung Medical Center Seoul Republic of Korea

**Keywords:** intensive care unit, machine learning, mechanical ventilator, patient safety, unplanned extubation

## Abstract

**Background:**

Patient safety in the intensive care unit (ICU) is one of the most critical issues, and unplanned extubation (UE) is considered the most adverse event for patient safety. Prevention and early detection of such an event is an essential but difficult component of quality care.

**Objective:**

This study aimed to develop and validate prediction models for UE in ICU patients using machine learning.

**Methods:**

This study was conducted in an academic tertiary hospital in Seoul, Republic of Korea. The hospital had approximately 2000 inpatient beds and 120 ICU beds. As of January 2019, the hospital had approximately 9000 outpatients on a daily basis. The number of annual ICU admissions was approximately 10,000. We conducted a retrospective study between January 1, 2010, and December 31, 2018. A total of 6914 extubation cases were included. We developed a UE prediction model using machine learning algorithms, which included random forest (RF), logistic regression (LR), artificial neural network (ANN), and support vector machine (SVM). For evaluating the model’s performance, we used the area under the receiver operating characteristic curve (AUROC). The sensitivity, specificity, positive predictive value, negative predictive value, and F1 score were also determined for each model. For performance evaluation, we also used a calibration curve, the Brier score, and the integrated calibration index (ICI) to compare different models. The potential clinical usefulness of the best model at the best threshold was assessed through a net benefit approach using a decision curve.

**Results:**

Among the 6914 extubation cases, 248 underwent UE. In the UE group, there were more males than females, higher use of physical restraints, and fewer surgeries. The incidence of UE was higher during the night shift as compared to the planned extubation group. The rate of reintubation within 24 hours and hospital mortality were higher in the UE group. The UE prediction algorithm was developed, and the AUROC for RF was 0.787, for LR was 0.762, for ANN was 0.763, and for SVM was 0.740.

**Conclusions:**

We successfully developed and validated machine learning–based prediction models to predict UE in ICU patients using electronic health record data. The best AUROC was 0.787 and the sensitivity was 0.949, which was obtained using the RF algorithm. The RF model was well-calibrated, and the Brier score and ICI were 0.129 and 0.048, respectively. The proposed prediction model uses widely available variables to limit the additional workload on the clinician. Further, this evaluation suggests that the model holds potential for clinical usefulness.

## Introduction

Patient safety in the intensive care unit (ICU) is a critical issue. Medical errors and adverse events can significantly impact patient outcomes [1]. Medical errors are a common occurrence in the ICU and airway-related accidents are the most frequent [2]. Adverse events related to airway and mechanical ventilation, such as unplanned extubation (UE), may lead to high rates of morbidity and mortality [3].

UE is a critical adverse event in the ICU, necessitating immediate action and treatment by the medical staff. In the literature, UE incidence rates range from 0.5 to 35.8 per 100 ventilated patients [4,5]. Previous studies have revealed that UE is associated with significant complications, such as airway injury, prolonged respiratory distress, aspiration, and hypoxemia [6]. Even after reintubation, UE remains associated with longer ICU stays [7] and an increased risk of ventilator-associated pneumonia [8].

Strategies to prevent UE include introducing a quality improvement program and novel devices [9,10]. However, for effective application of these tools, continuous screening and early detection is necessary. An electronic health record (EHR)-based prediction system could be an efficient and timely tool to provide continuous screening and early detection.

The wide establishment of advanced EHR systems has facilitated the development of machine learning prediction models [11]. These systems have shown substantial potential in predicting complex clinical conditions, such as sepsis, readmission, and cardiopulmonary resuscitation [12-14]. However, we were unable to find published examples of machine learning prediction models that were used for UE prediction. Therefore, the objective of this study was to develop and validate machine learning–based UE prediction models for patients in the ICU.

## Methods

The Transparent Reporting of a multivariable prediction model for Individual Prognosis Or Diagnosis (TRIPOD) statement [15] was followed for reporting our multivariable prediction model.

### Study Setting and Data Source

A single-center, retrospective study was conducted based on the EHR data of an academic tertiary hospital in Seoul, Republic of Korea. Data from January 2010 to December 2018 were extracted from the clinical data warehouse of the hospital, which contained deidentified clinical data for research. The hospital has approximately 2000 inpatient beds and 120 ICU beds. There are two types of ICUs: a medical ICU and a surgical ICU. In this study, 42 beds for the medical ICU and 70 beds for the surgical ICU were included. As of January 2019, there were approximately 9000 patients in the outpatient department and 250 patients in the emergency department on a daily basis. The number of annual ICU admissions is approximately 10,000.

### Study Population

The study population included patients who underwent extubation in the ICU between January 1, 2010, and December 31, 2018. Patients under the age of 18 years and patients who had multiple extubation episodes were excluded from the study. Patients who had been on mechanical ventilation for less than 24 hours or for more than 2 weeks were also excluded: patients with short mechanical ventilation periods had been admitted to the ICU only for a short period of observation, and the ICU protocol was to perform tracheostomy on patients by 2 weeks from the intubation.

### Outcome of Prediction Models

The risk prediction models used in this study had binary outcomes. They dealt with either the occurrence or absence of UE for an intubated ICU patient based on data from the last 8 hours.

### Data Set

We constructed a data set containing UE risk factors based on a literature review, which included the following: Confusion Assessment Method for the ICU (CAM-ICU) [16], the Richmond Agitation-Sedation Scale (RASS) [17], the Glasgow Coma Scale (GCS), upper-limb motor power, lower-limb motor power, the use of physical restraints, and work shifts. Because intubated patients cannot be assessed through verbal response due to the presence of an artificial airway, the verbal response records in the GCS were not considered. All included variables were routinely recorded by a nurse in the critical care flow sheet in the ICUs. The patients’ baseline characteristics were also included in the data set, consisting of age, sex, whether the patient underwent surgery prior to ICU admission, intubation location, and reason for ICU admission.

We split the data sets periodically for development and validation. The data sets acquired between January 1, 2010, and December 31, 2015, were used for development sets. The data sets acquired between January 1, 2016, and December 31, 2018, were used for validation sets.

### Data Preprocessing

#### Time-Window Setting

Features related to the CAM-ICU, the RASS, the GCS, and limb motor powers changed over time in the data sets. We set up a time window to consider the changing trends over time in these time-series features. We calculated the average recording intervals for each time-series feature and set 8 hours as the size of our time window, which covered the longest interval among them; as such, we expected that at least one change for all time-series features would be considered in the 8-hour time window. In addition, the characteristics of the clinical workflow of the institution were reflected. In the ICU where the study was conducted, nurses usually worked three shifts. We considered the time point at which the change in the patient’s condition could be sufficiently reflected in the EHR and, finally, an 8-hour window was selected.

#### Defining Cases and Controls

**Figure 1:**
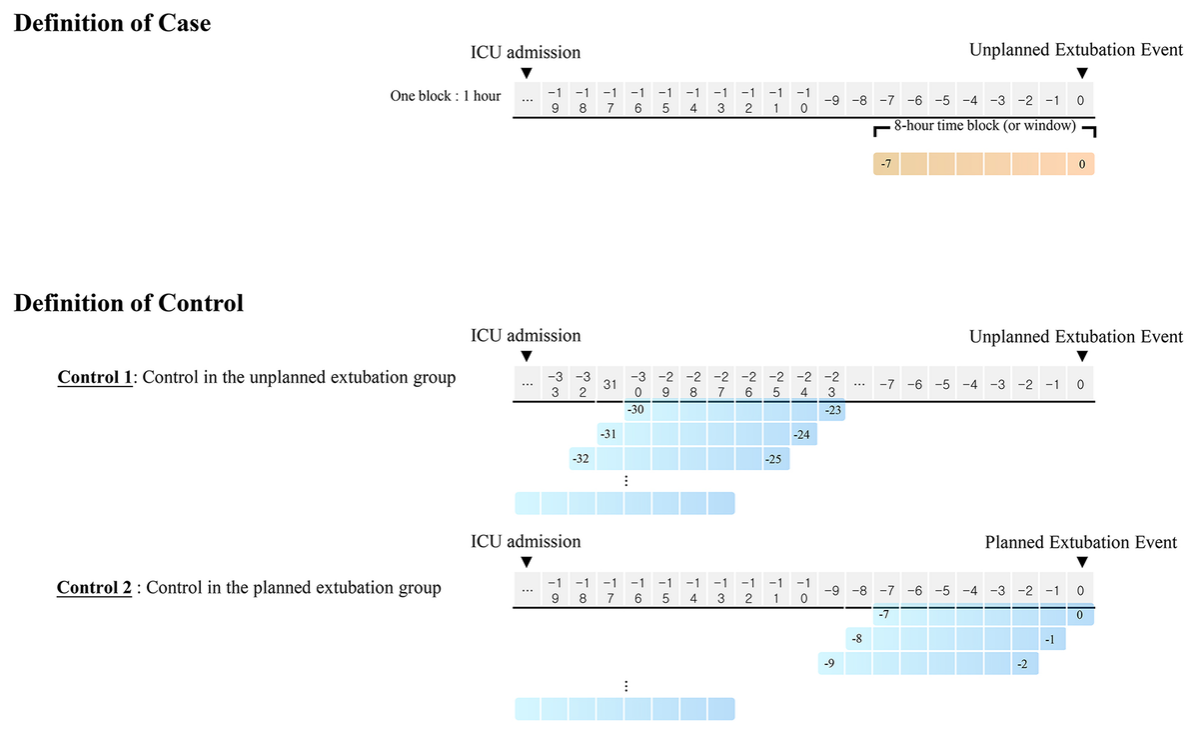
Case and control definitions using the time window in the time-series data set. ICU: intensive care unit.

A moving window with an 8-hour period was used to define cases and controls. The case and control definitions using the time window in the time-series data set is shown in Figure 1. When the UE event occurred, the 8-hour time block, or window, was annotated as a case. The 8-hour time block from ICU admission to 24 hours prior to the UE event (control 1) and the 8-hour time block from ICU admission to planned extubation event (control 2) were annotated as a control.

#### Time-Series Feature Handling

Time-series features were preprocessed to derive the representative values within an 8-hour time window. The values recorded closest to the specific time point and the recording frequencies over 8 hours prior to the time point were used as the representative values. In addition, the maximum, minimum, mean, and standard deviation values over 8 hours were calculated for numerical features (eg, the RASS, the GCS, and limb motor powers), and the recording frequencies for each category over 8 hours were considered for categorical features (eg, the CAM-ICU). We normalized the range of numerical features using a standardization method, which makes them have zero-mean and unit variance. We computed the parameters for normalization in the development sets and applied them to the full data sets.

#### Undersampling in the Data Sets

The number of UEs was scarce compared to planned extubation, resulting in an imbalance between the case and control numbers. To prevent overfitting of the control data, we undersampled the control 1 group using a simple random-sampling method and the control 2 group (ie, data from the planned extubation group) using a proportional stratified-sampling method. The days when the UE patients were on mechanical ventilation in the data sets were categorized into four groups. Control 2 data were sampled to thrice that of case data, while preserving the same proportion of days on mechanical ventilation for UE patients, as shown in Table 1. The sampled control data were independent, and the ratio of case to control 1 to control 2 in the data sets was approximately 1:1:3.

**Table 1:** Detailed information about unplanned extubation (UE) patients on mechanical ventilation that was used when undersampling the control 2 group.

Days on mechanical ventilation for UE patients^a^	Value (n=248), n (%)
1-2 days	83 (33.5)
2-3 days	53 (21.4)
3-5 days	55 (22.2)
>5 days	57 (23.0)

^a^Days when UE patients were on mechanical ventilation in the data sets were categorized into four groups.

#### Handling of Missing Data

We excluded 0.35% of the data where the RASS, the GCS, and limb motor powers were not recorded at least once in the whole time-series data sets. In terms of the features, the nearest value of the CAM-ICU was missing when there was no CAM-ICU record after ICU admission, where the missing rate was 33.46%. The missing data were assessed as *missing not at random* because the CAM-ICU was introduced to the hospital in which the study was conducted in late 2011 [18]. The CAM-ICU data were available after the method was introduced to the hospital, and there were many missing data at the beginning. We treated these data as a separate category altogether [19]. No missing data were estimated in the other features.

### Feature Selection

Backward elimination, a stepwise approach, was used for feature selection. The random forest (RF) algorithm was applied to all the features, and the least important features, based on the measured predictor importance, were excluded [20]. Finally, a subset of features that optimized area under the receiver operating characteristic curve (AUROC) values was selected to develop the UE prediction models. AUROC scores that were based on varying numbers of features selected are shown in Multimedia Appendix 1. A total of 50 selected features as input of the models and their importance values are shown in Multimedia Appendix 2. The features and their importance values are plotted in Multimedia Appendix 3.

### Modeling

#### Machine Learning Models

The following models were used to develop the UE prediction models: support vector machine (SVM), artificial neural network (ANN), logistic regression (LR), and RF [21-24].

#### Parameter Tuning

The parameters for SVM with the radial basis function kernel, LR, and RF models were tuned using grid search processes in the development sets, where the parameters with the best AUROC performance were selected. The hyperparameters for ANN, such as the number of layers and nodes in each layer, were tuned empirically. We used a five-layer network, with hidden layers having three to five times more neurons compared to the input features. For the activation function, a rectified linear unit was used in the hidden layer and a sigmoid function was used for the output layer [25]. To prevent the ANN from overfitting, we applied L2 regularization and dropout regularization [26,27]. The network was trained using mini-batch gradient descent and optimized using the cross-entropy method [28,29].

### Validation

Initially, we conducted internal validation on the development sets to quantify optimism in the predictive performance and evaluate stability of the prediction model. Three repeated and stratified 5-fold cross-validation techniques were used to evaluate the internal validity of each model. In brief, the data set was randomly divided into five parts of roughly equal size, while preserving the ratio of cases and controls. When one part was used for validation, the remaining four parts were used for model training, where each prediction was summarized into the AUROC. This procedure, as mentioned above, was repeated three times.

Prior to validating the machine learning models based on the validation sets, thresholds for each model were determined. Three repeated and stratified 5-fold cross-validations were used in the development sets to identify the best threshold. The mean of 15 sensitivities and the mean of 15 specificities were calculated at thresholds from 0 to 1 with 0.005 units. The selected thresholds for each model had a mean sensitivity over 0.85, and the best threshold was identified to be the one with the highest mean specificity. Finally, the models were applied to the validation sets.

### Statistical Analysis

Continuous variables were reported either as means and SDs for normal distribution data or as medians and IQRs for nonnormal distribution data. Categorical variables were reported as frequencies and percentages. We used the *t* test, the chi-square test, and the Wilcoxon rank-sum test to calculate the *P* values between the groups, where *P*<.05 was considered statistically significant.

The internal validation performance was evaluated through means and 95% CIs of the AUROCs. The performance of each model on the validation sets was evaluated with the AUROC, along with sensitivity, specificity, negative predictive value (NPV), positive predictive value (PPV), and the F1 score at the selected threshold.

For performance evaluation of the prediction model, we used a calibration curve, the Brier score [18,30], and the integrated calibration index (ICI) [31]. The potential clinical usefulness of the final model at the best threshold was assessed through a net-benefit approach using a decision curve [32]. This helps in determining if basing clinical decisions on a model is recommended considering the harm that it might cause, if any, in clinical practice. For statistical analyses and modeling, R, version 3.6.0 (The R Foundation) [33], and Python, version 3.6.6 (Python Software Foundation), were used [34]. The codes for developing and validating the models are available online [35].

### Sample Size

The data sample for a diagnostic model should have an appropriate size [36]. Since there was no previous study that could directly be referred to, this study followed an often-used “rule of thumb,” where the sample size ensured at least 10 events per candidate predictor parameter [37,38]. The number of presumed events per candidate predictor in this study was 15, satisfying the rule.

### Ethics Approval

The Institutional Review Board (IRB) of Samsung Medical Center approved this study (IRB file No. 2019-09-025).

## Results

### Study Population

**Figure 2:**
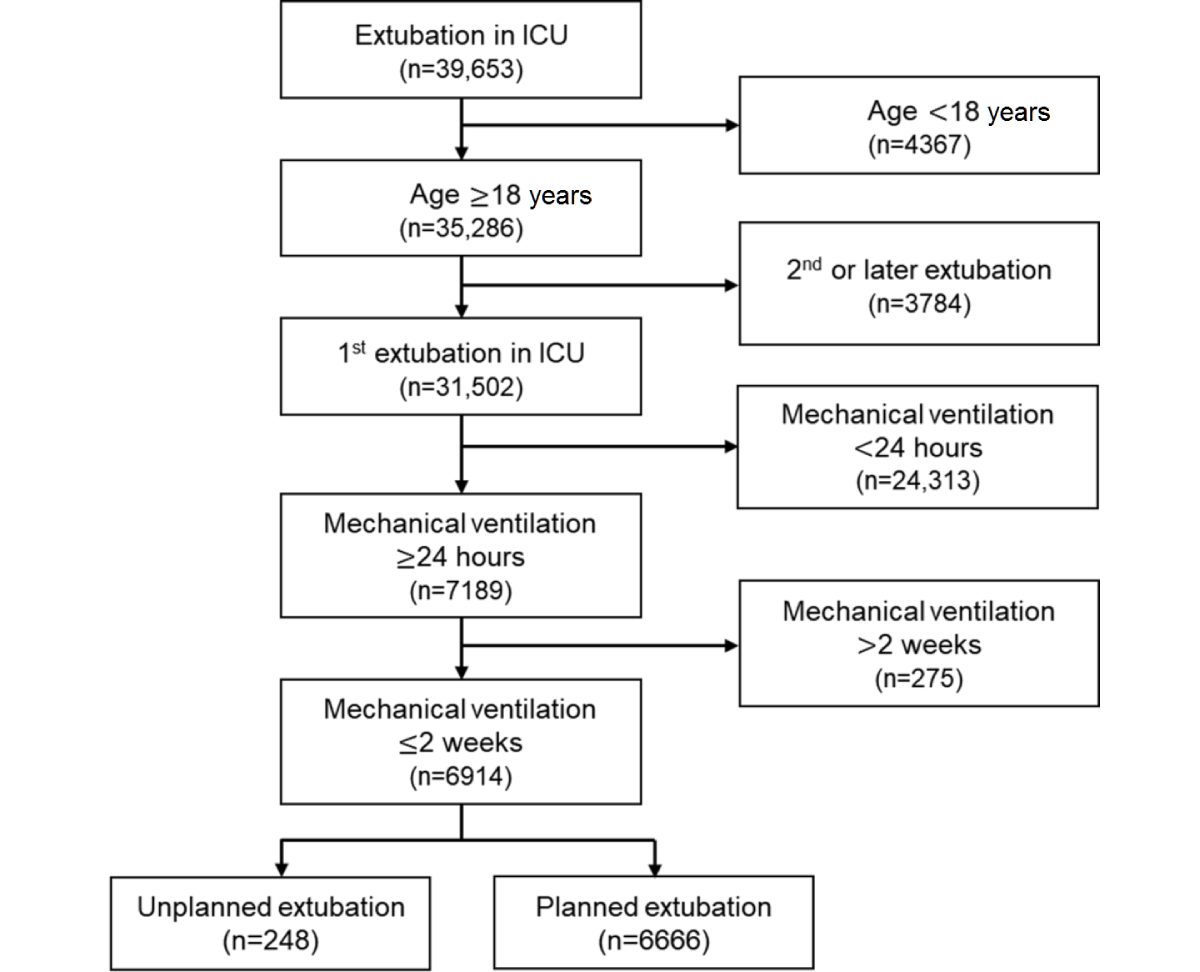
Flow diagram of the participant selection process. ICU: intensive care unit.

A total of 6914 extubation cases that had occurred between January 1, 2010, and December 31, 2018, were included in the study. The flow diagram of the participant selection process is shown in Figure 2.

The basic characteristics of the included cases are listed in Table 2. During the study period, the occurrence of 248 UEs were reported. There were more males than females in the UE group. The UE group also had fewer surgical patients and a high proportion of patients with physical restraints. Both ICU mortality and hospital mortality were significantly higher in the UE group than in the planned extubation group. Further, the rate of reintubation within 24 hours was higher in the UE group. However, no differences were noted between groups regarding the length of mechanical ventilation.

Table 3 lists the characteristics of the development and validation sets. In the case group, where a UE event occurred, the recording frequency of the RASS over the last 8 hours, a RASS score over 2, eye and motor responses of the GCS, upper-limb motor power, lower-limb motor power, and the rate of physical restraint use were higher than in the control group for both the development and validation sets. The *missing rate* of CAM-ICU data in the validation sets was noticeably lower than in the development sets.

**Table 2:** Basic characteristics and outcomes of the study population.

Characteristics and outcomes		Unplanned extubation (n=248)	Planned extubation (n=6666)	*P* value	
Age (years), mean (SD)		62.2 (13.8)	62.1 (14.9)	.97	
**Sex, n (%)**					<.001
	Male	190 (76.6)	4319 (64.8)		
	Female	58 (23.4)	2347 (35.2)		
**Cardiopulmonary resuscitation, n (%)**					.32
	No	241 (97.2)	6377 (95.7)		
	Yes	7 (2.8)	289 (4.3)		
**Surgery, n (%)**					<.001
	No	184 (74.2)	3471 (52.1)		
	Yes	64 (25.8)	3195 (47.9)		
**Intubation location, n (%)**					<.001
	Emergency room	33 (13.3)	611 (9.2)		
	Intensive care unit (ICU)	176 (71.0)	3997 (60.0)		
	Operating room	17 (6.9)	1298 (19.5)		
	Ward or others	22 (8.9)	760 (11.4)		
**Reason for ICU admission, n (%)**					<.001
	Respiratory	138 (55.6)	2459 (36.9)		
	Cardiovascular	41 (16.5)	909 (13.6)		
	Perioperative	38 (15.3)	2345 (35.2)		
	Others	31 (12.5)	953 (14.3)		
**Use of physical restraint, n (%)^a^**					<.001
	No	96 (38.7)	4275 (64.1)		
	Yes	152 (61.3)	2391 (35.9)		
**Work shift, n (%)**					<.001
	Day (7 AM to 3 PM)	94 (37.9)	4121 (61.8)		
	Evening (3 PM to 11 PM)	62 (25.0)	2123 (31.8)		
	Night (11 PM to 7 AM)	92 (37.1)	422 (6.3)		
**ICU mortality, n (%)**					<.001
	No	198 (79.8)	5847 (87.7)		
	Yes	50 (20.2)	819 (12.3)		
**In-hospital mortality, n (%)**					<.001
	No	150 (60.5)	4792 (71.9)		
	Yes	98 (39.5)	1847 (28.1)		
**Reintubation within 24 hours, n (%)**					<.001
	No	149 (60.1)	6128 (91.9)		
	Yes	99 (39.9)	538 (8.1)		
Mechanical ventilation days, median (IQR)		2.7 (3.3)	2.9 (4.0)	.17	
Hospital days, median (IQR)		27.5 (32.3)	25.0 (33.9)	.29	

^a^Use of physical restraint indicates whether physical restraint was applied in a case when extubated.

**Table 3:** Characteristics of the development and validation sets.

Characteristics^a^			Development sets (n=1004)				Validation sets (n=191)		
			Case (n=209)		Control (n=795)		Case (n=39)		Control (n=152)
Age (years), mean (SD)			61.43 (13.86)		61.85 (14.39)		66.10 (13.13)		63.71 (14.97)
**Sex, n (%)**									
	Male	159 (76.1)		522 (65.7)		31 (79.5)		100 (65.8)	
	Female	50 (23.9)		273 (34.3)		8 (20.5)		52 (34.2)	
**Surgery, n (%)**									
	No	52 (24.9)		294 (37.0)		5 (12.8)		30 (19.7)	
	Yes	157 (75.1)		501 (63.0)		34 (87.2)		122 (80.3)	
**Intubation location, n (%)**									
	Emergency room	26 (12.4)		61 (7.7)		7 (17.9)		24 (15.8)	
	Intensive care unit (ICU)	149 (71.3)		541 (68.1)		27 (69.2)		107 (70.4)	
	Operating room	15 (7.2)		94 (11.8)		2 (5.1)		13 (8.6)	
	Ward or others	19 (9.1)		99 (12.5)		3 (7.7)		8 (5.3)	
**Reason for ICU admission, n (%)**									
	Respiratory	36 (17.2)		237 (29.8)		2 (5.1)		24 (15.8)	
	Cardiovascular	30 (14.4)		108 (13.6)		11 (28.2)		29 (19.1)	
	Perioperative	36 (17.2)		237 (29.8)		2 (5.1)		24 (15.8)	
	Others	28 (13.4)		109 (13.7)		3 (7.7)		32 (21.1)	
**Recording frequency, mean (SD)**									
	Confusion Assessment Method for the Intensive Care Unit (CAM-ICU)	0.65 (0.63)		0.55 (0.53)		1.15 (0.43)		0.99 (0.45)	
	Richmond Agitation-Sedation Scale (RASS)	3.75 (5.93)		2.02 (2.84)		3.69 (3.64)		2.28 (2.72)	
	Glasgow Coma Scale (GCS)	3.38 (1.93)		3.52 (2.16)		2.59 (0.85)		2.91 (1.74)	
	Upper-limb motor power	3.01 (1.79)		3.18 (2.19)		2.54 (1.05)		2.79 (1.77)	
	Lower-limb motor power	3.01 (1.79)		3.17 (2.19)		2.51 (1.05)		2.79 (1.77)	
	Use of physical restraint	1.00 (1.07)		0.61 (0.84)		0.95 (0.69)		0.59 (0.67)	
**Nearest value of CAM-ICU, n (%)**									
	Negative	49 (23.4)		148 (18.6)		14 (35.9)		48 (31.6)	
	Positive	59 (28.2)		151 (19.0)		24 (61.5)		57 (37.5)	
	Unable to access	18 (8.6)		135 (17.0)		1 (2.6)		38 (25.0)	
	Missing	83 (39.7)		361 (45.4)		0 (0)		9 (5.9)	
**Nearest value of RASS, n (%)**									
	less than –2	20 (9.8)		213 (26.9)		2 (5.6)		42 (28.2)	
	–2 or –1	31 (15.1)		166 (20.9)		7 (19.4)		43 (28.9)	
	0	51 (24.9)		190 (24.0)		9 (25.0)		26 (17.4)	
	+1 or +2	60 (29.3)		163 (20.6)		4 (11.1)		25 (16.8)	
	more than +2	43 (21.0)		61 (7.7)		14 (38.9)		13 (8.7)	
**Nearest value of GCS, mean (SD)**									
	Eye response	3.38 (0.93)		2.93 (1.15)		3.54 (0.68)		3.04 (1.08)	
	Motor response	5.49 (1.19)		4.86 (1.71)		5.79 (0.52)		5.05 (1.60)	
**Nearest value of upper-limb motor power, mean (SD)**									
	Right	3.70 (1.31)		3.04 (1.62)		4.00 (0.76)		2.99 (1.59)	
	Left	3.72 (1.26)		3.05 (1.62)		4.10 (0.60)		3.00 (1.59)	
**Nearest value of lower-limb motor power, mean (SD)**									
	Right	3.00 (1.48)		2.59 (1.62)		3.44 (1.05)		2.59 (1.54)	
	Left	3.03 (1.48)		2.62 (1.61)		3.44 (1.05)		2.62 (1.56)	
**Nearest value of use of physical restraint, n (%)**									
	No	84 (40.2)		460 (57.9)		13 (33.3)		79 (52.0)	
	Yes	125 (59.8)		335 (42.1)		26 (66.7)		73 (48.0)	
**Work shift, n (%)**									
	Day (7 AM to 3 PM)	78 (37.3)		296 (37.2)		16 (41.0)		61 (40.1)	
	Evening (3 PM to 11 PM)	49 (23.4)		242 (30.4)		13 (33.3)		38 (25.0)	
	Night (11 PM to 7 AM)	82 (39.2)		257 (32.3)		10 (25.6)		53 (34.9)	

^a^For time-series features, the recording frequency over 8 hours prior to the time point and the nearest value to the time point were derived.

### Model Development and Assessment

A total of 50 features, selected through a recursive feature-elimination technique among the 66 candidates, reflected demographic characteristics and patterns of change in the time-series data. The features, their importance scores, and their variable types are listed in Multimedia Appendix 2. The list of the selected features with their corresponding importance scores are plotted in Multimedia Appendix 3.

We developed machine learning–based prediction algorithms using RF, LR, ANN, and SVM. The average AUROCs and 95% CIs for internal validation in the development sets were 0.732 (95% CI 0.705-0.759) for RF, 0.703 (95% CI 0.676-0.730) for LR, 0.670 (95% CI 0.637-0.702) for ANN, and 0.689 (95% CI 0.668-0.710) for SVM.

**Figure 3:**
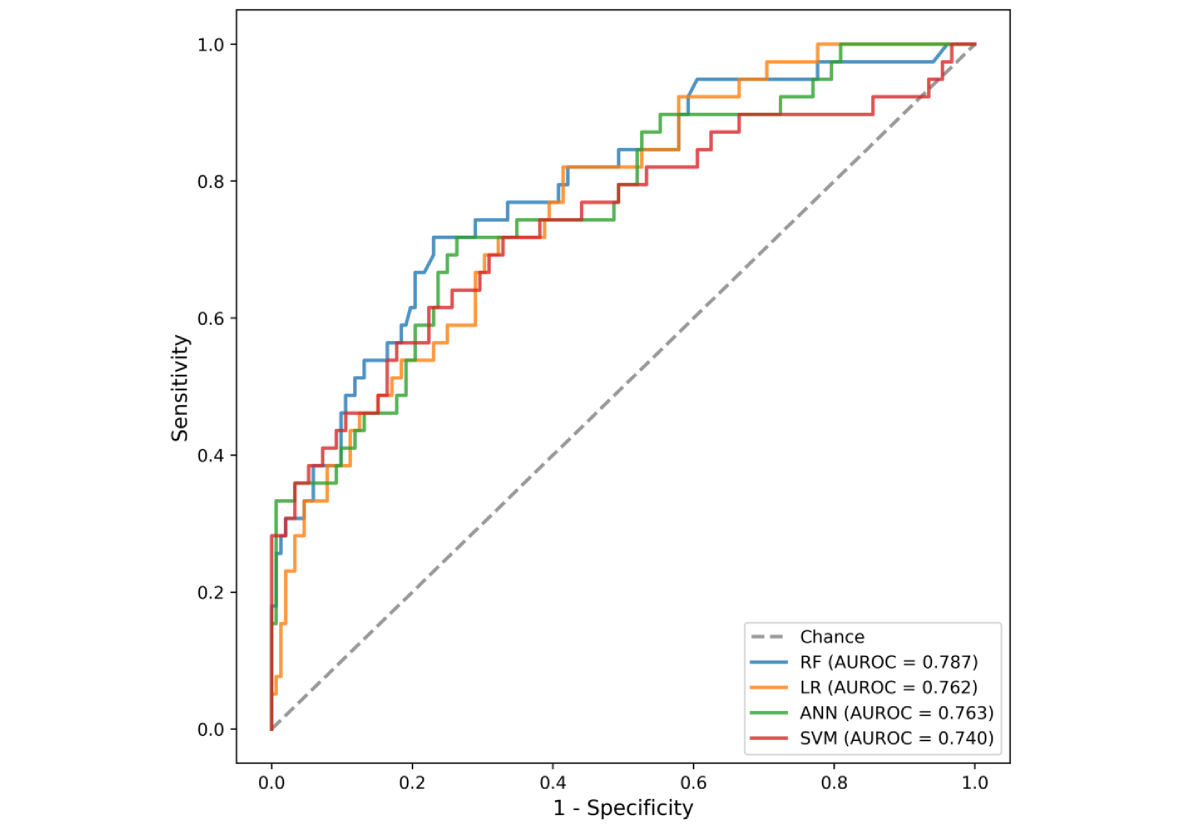
Receiver operating characteristic curves for all of the unplanned extubation prediction models. ANN: artificial neural network; AUROC: area under the receiver operating characteristic curve; LR: linear regression; RF: random forest; SVM: support vector machine.

For each model, the highest value of specificity among the sensitivities over 0.85 was selected as the cutoff point of the threshold. In terms of the machine learning models, the best model was RF, with the highest performance values at the selected threshold, where AUROC was 0.787 and sensitivity, specificity, NPV, PPV, F1 score, and ICI were 0.949, 0.388, 0.967, 0.285, 0.438, and 0.048, respectively. The performance values of the prediction models are listed in Table 4. The models’ AUROCs are shown in Figure 3.

**Figure 4:**
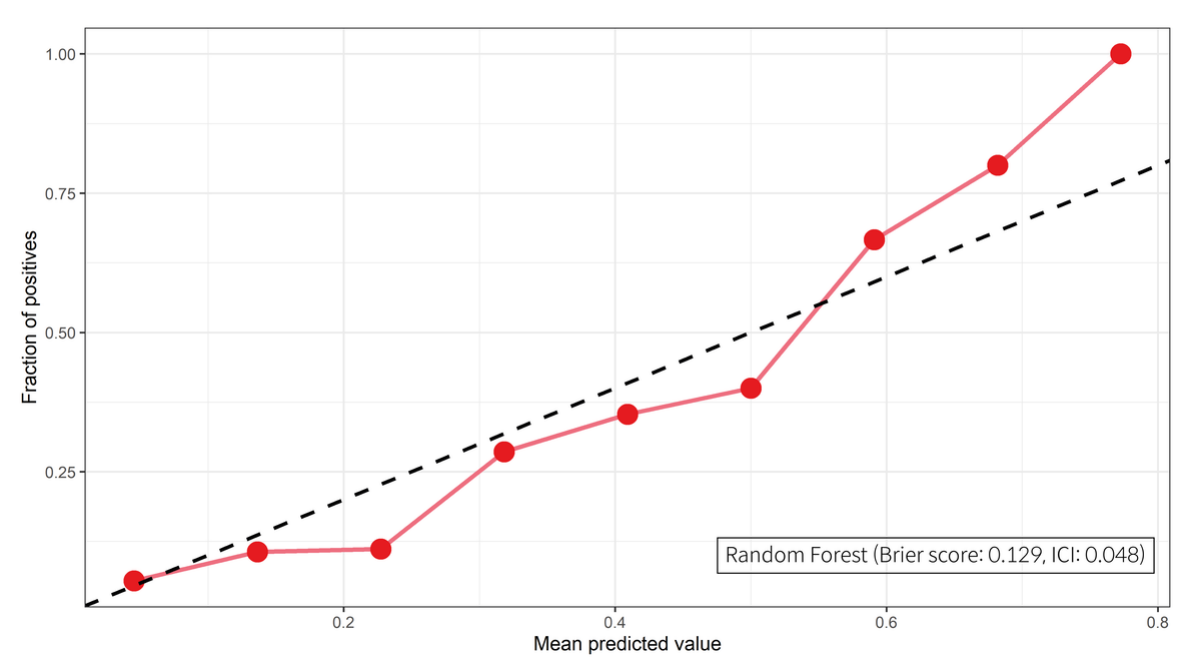
The calibration curve and the integrated calibration index (ICI) of the best model.

**Figure 5:**
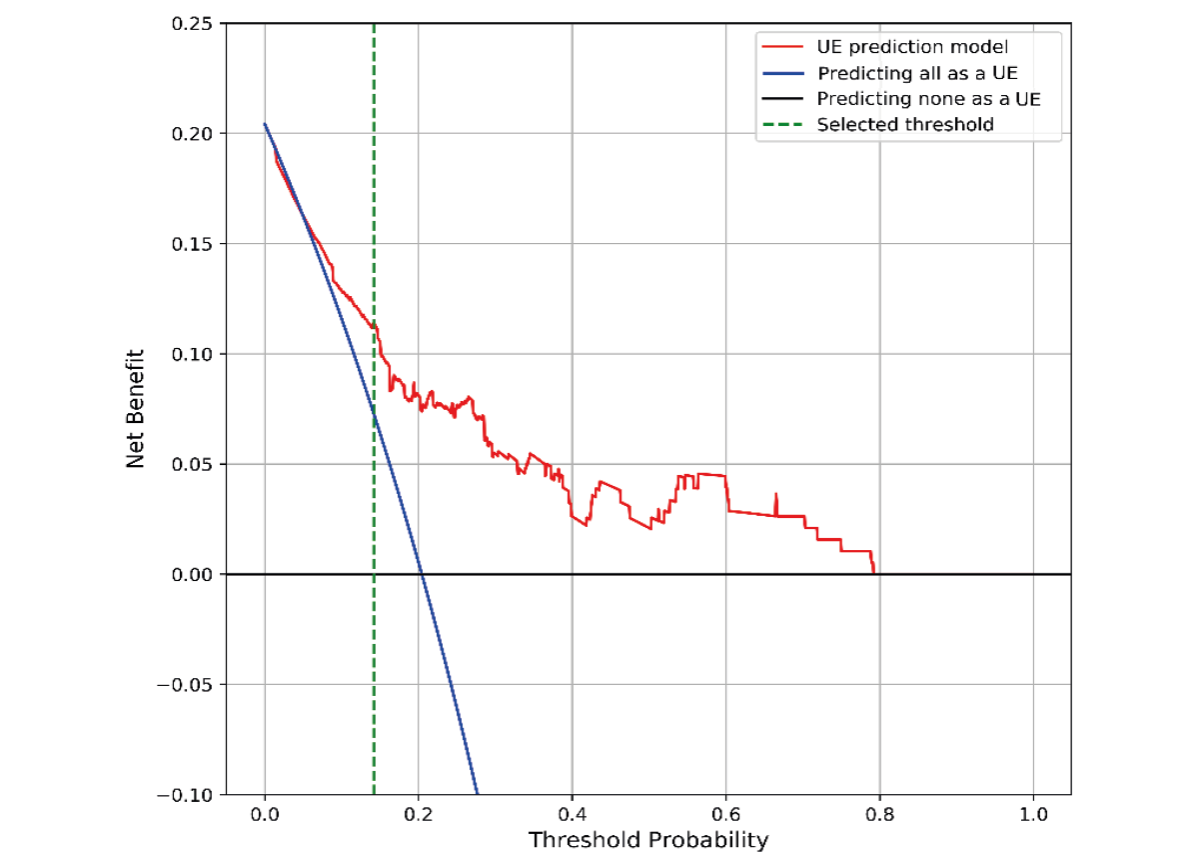
The decision curve of the best model. UE: unplanned extubation.

The performance of the best model was evaluated using the Brier score, the ICI, and decision curve analysis. The calibration, agreement between observed outcomes and predicted risk probabilities, was assessed with the slope of the calibration curve and the Brier score. The RF model was well-calibrated, and the Brier score and ICI were 0.129 and 0.048, respectively. The calibration curve of the best model is shown in Figure 4. The decision curve compared the net benefit of the best model and alternative approaches for clinical decision making. The decision curve showed superior net benefit when the best model was used compared to the alternative approaches of “predicting all as a UE” or “predicting none as a UE” over a threshold probability range of 6% to 78%. Our selected threshold was 14%, and it showed potentially superior clinical utility. The decision curve of the best model is presented in Figure 5.

**Table 4:** Comparison of performance values of the prediction models.

Model	AUROC^a^	Sensitivity	Specificity	NPV^b^	PPV^c^	F1 score	ICI^d^
Random forest	0.787	0.949	0.388	0.967	0.285	0.438	0.048
Linear regression	0.762	0.949	0.303	0.958	0.259	0.407	0.025
Artificial neural network	0.763	0.949	0.230	0.946	0.240	0.383	0.077
Support vector machine	0.740	0.897	0.283	0.915	0.243	0.383	0.050

^a^AUROC: area under the receiver operating characteristic curve.

^b^NPV: negative predictive value.

^c^PPV: positive predictive value.

^d^ICI: integrated calibration index.

## Discussion

### Principal Findings

For patient safety, prevention and early detection of clinical error is an essential component of high-quality care [1]. The proposed prediction model is expected to screen and monitor ICU patients effectively when applied to the clinical setting. To the best of our knowledge, this is the first machine learning–based prediction model for UE incidents, and it is an algorithm that predicts the UE within 1 hour, allowing clinical staff to take appropriate action to prevent UE. In the previous study, a simple LR-based statistical model was presented where the data were not divided into training and test sets [39].

The limitation of the machine learning prediction model is related to its ability to exhibit good performance in a real clinical setting. Our study assessed the performance of the UE prediction model; the best model demonstrated good calibration and net benefit over a wide range of threshold probabilities. This prediction model shows potentially superior clinical utility based on decision curve analysis [40].

### Comparison With Prior Work

Existing UE risk assessment tools and applications will have a limited impact if they include additional work for the nurses, such as requiring additional assessments or documentation tasks. An EHR-based prediction algorithm can automatically calculate the risk for clinical staff without any additional workload.

Alarm fatigue in the ICU is another major concern that disrupts the workflow of the clinician and can significantly impact patient safety [41]. The UE prediction model is intended to be used as a screening tool for predicting potential UE events, otherwise the false alarm rate would be high due to the low specificity and PPV [42]. Therefore, clinician stakeholders would need to be engaged in identifying ways to ensure that the alert is integrated into the clinical workflow in a way that is actionable. Clinicians should also be involved in setting appropriate threshold values based on their practice, workflow, and purpose for adopting the algorithm [43].

In previous studies, agitation was the most important factor among patient-associated risk factors for UE incidence. The incidence rate of UE varies according to the patient’s level of consciousness, recording frequency, and age; in addition, physical restraints were significant risk factors for UE (Multimedia Appendix 2). Recording frequency is presented as an important feature, and frequent recording of the patient’s condition in clinical practice provides an interpretation that improves predictions.

Further, this study revealed that the use of physical restraints was higher in the UE group. Though physical restraints are frequently used in ICUs to prevent UE [44,45], it can increase the risk of UE [46]. A factor that can be attributed to this ironic result is the use of restraints evoking delirium, which is related to self-extubation [47]. However, the physical restraints may have been warranted as a safety measure, but insufficiently applied and, therefore, unable to prevent UE.

### Limitations

This study was retrospective and carried out in a single center. To improve the model’s performance and for precise comparison among machine learning–based models, comparatively large clinical data sets and multicenter validation are required. All developed models seemed to have similar performances, assuming that small evaluation data sets caused this. Further, prospective studies are required to verify the algorithm’s performance.

There are limitations in terms of the number of small data sets and random sampling for the control 2 group, resulting in a biased sample. Although UE is a significant complication in the ICU, its incident rate was reported to be low in the previous studies. Thus, it is complicated to obtain large amounts of data on events related to patient safety accidents. Obtaining ample data is a crucial concern in machine learning. Validating a prediction model requires a minimum of 100 events and 100 nonevents; however, our validation data set did not include 100 events. Instead, our study had 15 events per candidate predictor in the development data set and satisfied the well-used “rule of thumb.” Nevertheless, machine learning is possible with the use of small data sets [48-50]. We conducted a stratified undersampling method to avoid overfitting, and data were sampled randomly. This method can potentially discard important information or results in a biased sample.

In this study, we included short-term mortality (ie, ICU mortality) and in-hospital mortality. We could not follow up on deaths of patients after discharge. Further, we have not considered long-term survival and correlation between comorbidity and duration of mechanical ventilation.

### Future Research

The models were developed retrospectively and carried out in a single center; therefore, future prospective evaluation and validation using other data sets are required.

### Conclusions

We developed a machine learning prediction model for UE patients. The best AUROC was 0.787, and the sensitivity was 0.949 at the selected threshold for the best model. The best model was well-calibrated, and the Brier score and ICI were 0.129 and 0.048, respectively. The proposed prediction model uses widely available variables to limit the additional workload on the clinician. Further, this evaluation suggests that the model holds potential for clinical usefulness.

## Appendix

Multimedia Appendix 1 Area under the receiver operator characteristic curve (AUROC) scores based on varying numbers of features selected. A stepwise backward elimination technique (recursive feature elimination [RFE])—based on feature importance derived from the random forest algorithm with 500 trees and three repeated and stratified 5-fold cross-validation techniques—was used to select the optimal subset of features. The feature subset scores were based on the mean of the AUROCs from cross-validation. The RFE with cross-validation (RFECV) function in the scikit-learn package, version 0.22.1, was used for feature selection.

Multimedia Appendix 2 The 50 selected features as input of the models.

Multimedia Appendix 3 Importance of features included in the unplanned extubation prediction models following application of the random forest algorithm.

## Abbreviations

ANN: artificial neural network
AUROC: area under the receiver operating characteristic curve
CAM-ICU: Confusion Assessment Method for the Intensive Care Unit
EHR: electronic health record
GCS: Glasgow Coma Scale
ICI: integrated calibration index
ICU: intensive care unit
IRB: Institutional Review Board
LR: logistic regression
NPV: negative predictive value
PPV: positive predictive value
RASS: Richmond Agitation-Sedation Scale
RF: random forest
SVM: support vector machine
TRIPOD: Transparent Reporting of a multivariable prediction model for Individual Prognosis Or Diagnosis
UE: unplanned extubation
