# Supplementary figures and images for: Development and Validation of Unplanned Extubation Prediction Models Using Intensive Care Unit Data: Retrospective, Comparative, Machine Learning Study
Source: J Med Internet Res. 2021 Aug 11;23(8):e23508. doi: 10.2196/23508 (PMC8387891; doi:10.2196/23508)

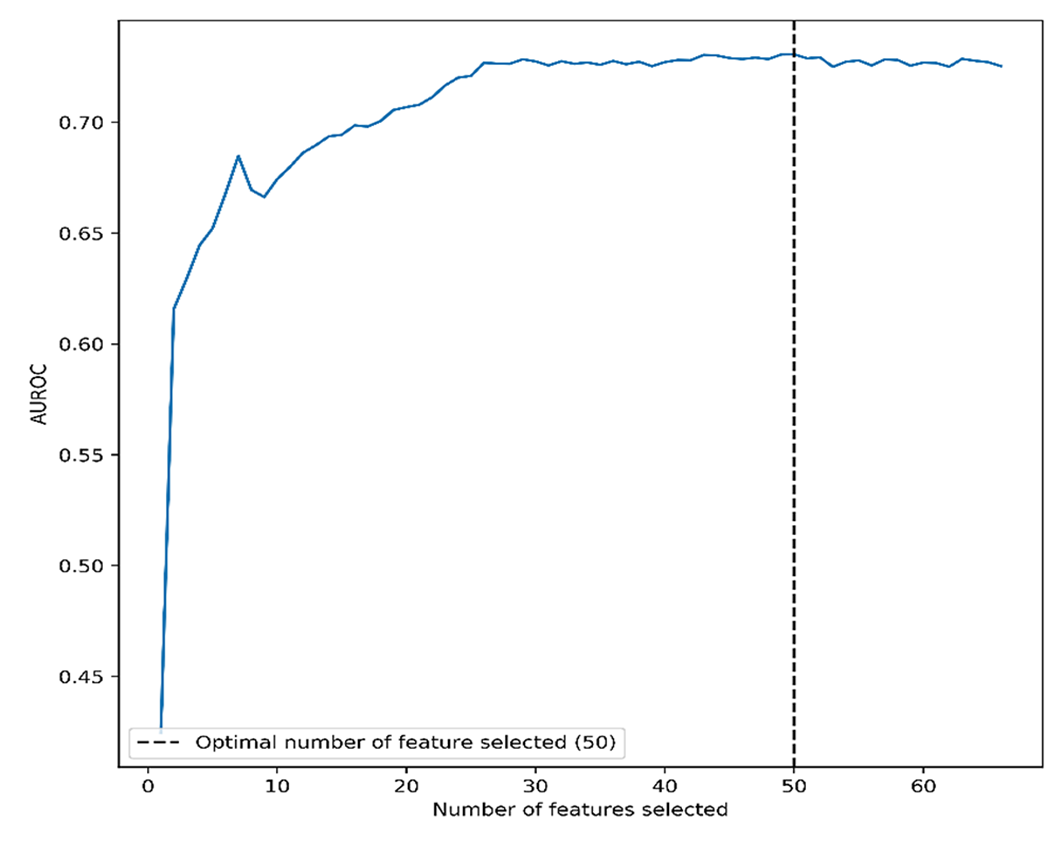

Supplement: Multimedia Appendix 1 [file jmir_v23i8e23508_app1.png]

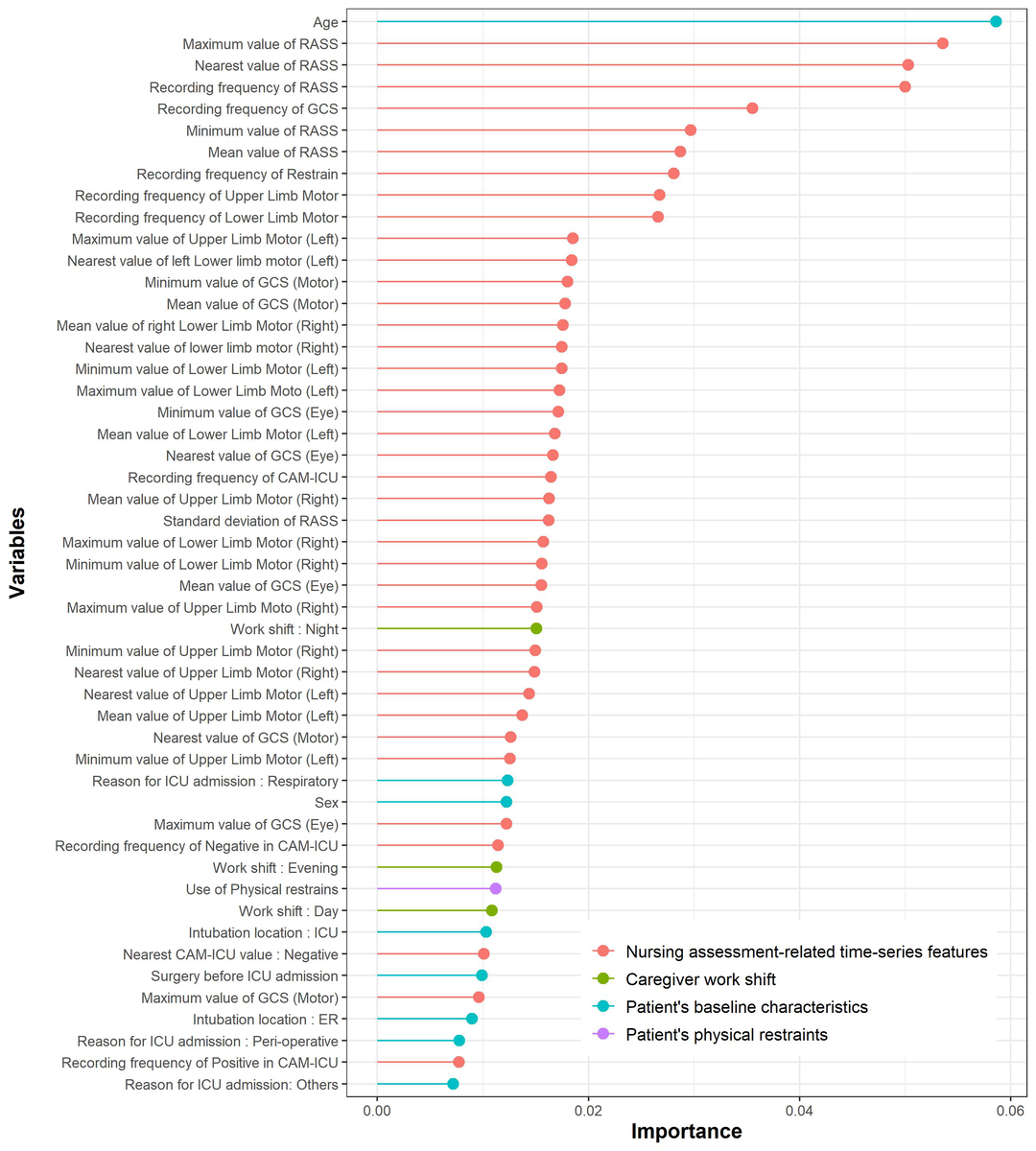

Supplement: Multimedia Appendix 3 [file jmir_v23i8e23508_app3.png]
