# Supplementary material for: Development and Validation of Unplanned Extubation Prediction Models Using Intensive Care Unit Data: Retrospective, Comparative, Machine Learning Study
Source: J Med Internet Res. 2021 Aug 11;23(8):e23508. doi: 10.2196/23508 (PMC8387891; doi:10.2196/23508)
Supplement: Multimedia Appendix 2 [file jmir_v23i8e23508_app2.docx]

**Multimedia Appendix 2.** 50 selected features as input of the models.

|  | Selected features | Feature importance | Variable type |
| --- | --- | --- | --- |
| 1 | Age | 0.058606392 | numerical |
| 2 | Maximum value of RASS | 0.053570709 | numerical |
| 3 | Nearest value of RASS | 0.050276102 | numerical |
| 4 | Recording frequency of RASS | 0.050002921 | numerical |
| 5 | Recording frequency of GCS | 0.0355196 | numerical |
| 6 | Minimum value of RASS | 0.02968151 | numerical |
| 7 | Mean value of RASS | 0.028696508 | numerical |
| 8 | Recording frequency of Restrain | 0.028088406 | numerical |
| 9 | Recording frequency of Upper Limb Motor | 0.026736112 | numerical |
| 10 | Recording frequency of Lower Limb Motor | 0.026593318 | numerical |
| 11 | Maximum value of Upper Limb Motor (Left) | 0.018507774 | numerical |
| 12 | Nearest value of left Lower limb motor (Left) | 0.018406376 | numerical |
| 13 | Minimum value of GCS (Motor) | 0.017993713 | numerical |
| 14 | Mean value of GCS (Motor) | 0.017804112 | numerical |
| 15 | Mean value of right Lower Limb Motor (Right) | 0.017581739 | numerical |
| 16 | Nearest value of lower limb motor (Right) | 0.017474889 | numerical |
| 17 | Minimum value of Lower Limb Motor (Left) | 0.017473873 | numerical |
| 18 | Maximum value of Lower Limb Moto (Left) | 0.017244662 | numerical |
| 19 | Minimum value of GCS (Eye) | 0.017133784 | numerical |
| 20 | Mean value of Lower Limb Motor (Left) | 0.016828286 | numerical |
| 21 | Nearest value of GCS (Eye) | 0.016633066 | numerical |
| 22 | Recording frequency of CAM-ICU | 0.016457955 | numerical |
| 23 | Mean value of Upper Limb Motor (Right) | 0.016264442 | numerical |
| 24 | Standard deviation of RASS | 0.016243504 | numerical |
| 25 | Maximum value of Lower Limb Motor (Right) | 0.015711368 | numerical |
| 26 | Minimum value of Lower Limb Motor (Right) | 0.015571962 | numerical |
| 27 | Mean value of GCS (Eye) | 0.015541704 | numerical |
| 28 | Maximum value of Upper Limb Moto (Right) | 0.015091225 | numerical |
| 29 | Work shift : Night | 0.015078522 | binary categorical |
| 30 | Minimum value of Upper Limb Motor (Right) | 0.014959628 | numerical |
| 31 | Nearest value of Upper Limb Motor (Right) | 0.014895209 | numerical |
| 32 | Nearest value of Upper Limb Motor (Left) | 0.014358047 | numerical |
| 33 | Mean value of Upper Limb Motor (Left) | 0.013726986 | numerical |
| 34 | Nearest value of GCS (Motor) | 0.012644344 | numerical |
| 35 | Minimum value of Upper Limb Motor (Left) | 0.01254895 | numerical |
| 36 | Reason for ICU admission : Respiratory | 0.012326171 | binary categorical |
| 37 | Sex | 0.01221975 | binary categorical |
| 38 | Maximum value of GCS (Eye) | 0.012219087 | numerical |
| 39 | Recording frequency of Negative in CAM-ICU | 0.011434241 | numerical |
| 40 | Work shift : Evening | 0.011301314 | binary categorical |
| 41 | Nearest value of Use of Physical restrains | 0.011227373 | numerical |
| 42 | Work shift : Day | 0.010844866 | binary categorical |
| 43 | Intubation location : ICU | 0.010320196 | binary categorical |
| 44 | Nearest CAM-ICU value : Negative | 0.010091001 | binary categorical |
| 45 | Surgery before ICU admission | 0.009922412 | binary categorical |
| 46 | Maximum value of GCS (Motor) | 0.009625162 | numerical |
| 47 | Intubation location : ER | 0.008966365 | binary categorical |
| 48 | Reason for ICU admission : Peri-operative | 0.007778941 | binary categorical |
| 49 | Recording frequency of Positive in CAM-ICU | 0.00772266 | numerical |
| 50 | Reason for ICU admission: Reasons other than respiratory, cardiovascular, and peri operative | 0.00719536 | binary categorical |
